# Influence of Artificial Sweetener on Human Blood Glucose Concentration

**DOI:** 10.1100/tsw.2007.228

**Published:** 2007-10-05

**Authors:** Ilse Skokan, P. Christian Endler, Beatrix Wulkersdorfer, Dieter Magometschnigg, Heinz Spranger

**Affiliations:** Interuniversity College for Health and Development Graz, Castle of Seggau, Austria

**Keywords:** sacchrose, artificial sweeteners, blood glucose concentration, Diabetes mellitus, weight control

## Abstract

Artificial sweeteners, such as saccharin or cyclamic acid are synthetically manufactured sweetenings. Known for their low energetic value they serve especially diabetic and adipose patients as sugar substitutes. It has been hypothesized that the substitution of sugar with artificial sweeteners may induce a decrease of the blood glucose. The aim of this study was to determine the reliability of this hypothesis by comparing the influence of regular table sugar and artificial sweeteners on the blood glucose concentration. In this pilot-study 16 patients were included suffering from adiposity, pre-diabetes and hypertension. In the sense of a cross-over design, three test trials were performed at intervals of several weeks. Each trial was followed by a test free interval. Within one test trial each patient consumed 150 ml test solution (water) that contained either 6 g of table sugar (“Kandisin”) with sweetener free serving as control group. Tests were performed within 1 hr after lunch to ensure conditions comparable to patients having a desert. Every participant had to determine their blood glucose concentration immediately before and 5, 15, 30 and 60 minutes after the intake of the test solution. For statistics an analysis of variance was performed. The data showed no significant changes in the blood glucose concentration. Neither the application of sugar (F_4;60_ = 1.645; p = .175) nor the consumption of an artificial sweetener (F_2.068;31.023_ = 1.551; p > .05) caused significant fluctuations in the blood sugar levels. Over a time frame of 60 minutes in the control group a significant decrease of the blood sugar concentration was found (F_2.457;36.849_ = 4.005; p = .020) as a physiological reaction during lunch digestion.

## INTRODUCTION

Artificial sweeteners, such as saccharin (E954) or cyclamic acid (E952) are synthetically manufactured sweetening, whose chemical structures do not show any similarity to carbohydrates. Sweeteners are biochemically and biologically inactive[1] and much sweeter than sacchrose (regular table sugar). Known as adjuvant to decrease the energetic input, they serve many diabetics as sugar substitutes. Tables 1 and 2 demonstrate the nutrition facts of sugar and "Kandisin", an artificial sweetener containing saccharin and cyclamic acid. Since the European Scientific Committee on Food (SCF)[1] authorizes sweetener in Europe as food additives with an assigned acceptable daily intake (ADI) value and used especially by patients suffering from diabetes mellitus and additionally by the adipose population[2].

Nevertheless, according to a group of scientists a higher weight gain has been determined in the population consuming artificial sweeteners compared to those applying non-artificial sugar (sacchrose)[3,4,5]. Due to these findings the efficiency of artificial sweeteners has been questioned.

It has been discussed that the substitution of sugar with artificial sweeteners may cause a so called “cephalic insulin reflex”, a psychosomatic reflex, which induces a decrease of the blood glucose and is interpreted by the hypothalamus as a sign of hunger (glucostatic hypothesis)[6]. As a consequence the body would be longing for a higher caloric intake.

The aim of this pilot study was to determine the reliability of this hypothesis by comparing the influence of regular table sugar and artificial sweeteners on the blood glucose concentration in humans.

**Table 1**
**Nutrition facts of sacchrose (table sugar)**

| | Servings per 100 g | Servings per 6 g |
|---|---|---|
| Calories | 1697 kJ (405 kcal) | 101.82 kJ (24.30 kcal) |
| Protein | 0 g | 0 g |
| Carbohydrates | 100 g | 6 g |
| Fat | 0 g | 0 g |

**Table 2**
**Nutrition facts of “Kandisin”**

| | Servings per 100 g | Servings per piece |
|---|---|---|
| Calories | 121 kJ (28.5 kcal) | 0.09 kJ (0.021 kcal) |
| Protein | 0 g | 0 g |
| Carbohydrates | 0 g | 0 g |
| Fat | 0 g | 0 g |

## METHODS

For this study we chose a triple cross-over-design. Three test trials were performed at intervals of several weeks. Each trial was followed by a testing free interval of one month. 16 patients (n=16) were included in this study, five women and 11 men, between 58 and 82 years old. All participants were overweight or adipose (BMI 24-36.1), suffered from hypertension and pre-diabetes, showing a fasting-blood sugar between 96 and 125 mg/dl.

Within one test trial each patient consumed 150 ml test solution (water) that contained either 6 g of table sugar (“Kandisin”) with sweetener free serving as control group. Tests were performed within one hour after lunch to ensure conditions comparable to patients having a desert. The patients had to determine their blood glucose concentration before and 5, 15, 30 and 60 minutes after the intake of the test solution. Therefore a blood drop (1 µl) was delivered from a fingertip and measured with the help of a commonly used blood sugar measuring system (ONE TOUCH ® Ultra, Life Scan). All data were collected in a measuring protocol. For the statistical analysis an analysis of variance was performed.

## RESULTS

Based on the collected data no significant changes in the blood glucose concentration could be reached within the different measuring time points. Neither the application of sugar ($F_{4;60}$ = 1.645; p = .175) nor the consumption of an artificial sweetener ($F_{2.068;31.023}$ = 1.551; p > .05) caused significant fluctuations in the patient blood sugar levels. Over a time frame of 60 minutes in the control group a significant decrease of the blood sugar concentration was found ($F_{2.457;36.849}$ = 4.005; p = .020) due to the physiological reaction of the human body. Figure 1 below demonstrates the blood sugar concentrations under each condition at given time points and illustrates the results on the basis of its curve progression.

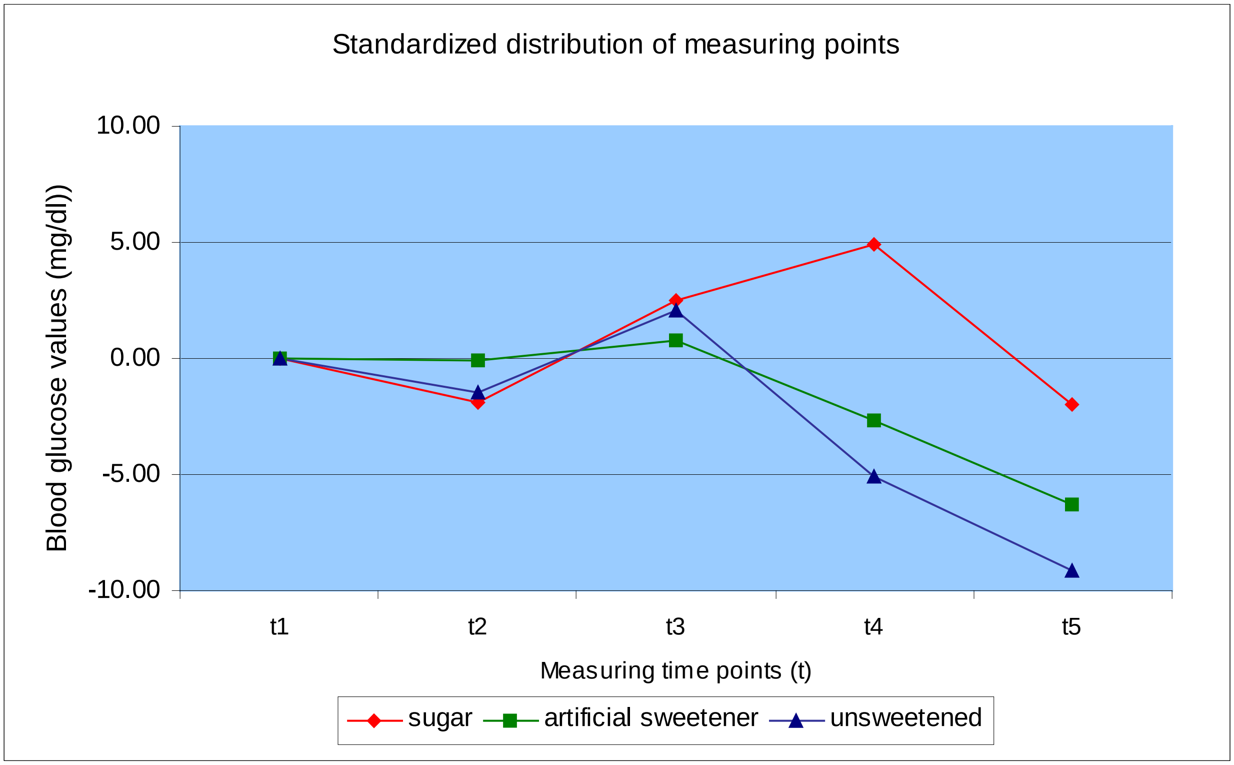

**Figure 1.** Standardized distribution of measuring points

## DISCUSSION

The aim of this study was to determine the impact of artificial sweeteners on the process of weigh loss. Main attention was drawn on the question if the intake of an artificial sweetener would cause an elevation of patient blood glucose concentration and furthermore a hunger-stimulation.

Considering the results in this pilot study it cannot be shown that artificial sweeteners may cause hunger stimulation. Artificial sweeteners as well as regular table sugar do not seem to have any influence on the patient blood sugar concentration. The only significant difference was determined in the trial without applying any kind of sweeteners. This development was expected as a physiological reaction.

Since insulin plays an important role in the regulation of the blood glucose concentration, the investigation of the socalled "cephalic insulin-reflex" should be considered by determining the connecting-peptide (C-peptide), which is secreted together with insulin. In this case an invasive intravenous blood analysis would have been required, which kept us from including this aspect in this study.

It is still unclear, whether the substitution of sacchrose with artificial sweeteners may have an important influence on the dietary food intake in our society.

Considering the epidemic spread, nutrition and life style related diseases, such as diabetes and adiposity, further studies demonstrate a big challenge and may have a high impact on our population.
